# The Cali Cancer Registry an example for Latin America

**Published:** 2012-12-30

**Authors:** Pelayo Correa

**Affiliations:** 1 Division of Gastroenterology, Department of Medicine, Vanderbilt University School of Medicine, Nashville, TN, USA. E-mail: pelayo.correa@vanderbilt.edu

The Cali Cancer Registry was started 50 years ago as a research program of the Department of Pathology of Universidad del Valle. It is presently continuing its strong silent but meaningful work, internationally recognized as the oldest most trustworthy and reliable source of data on descriptive epidemiology in Latin America. This is evidenced by the publication, of its results in "Cancer Incidence in Five Continents", the most prestigious source of data on cancer incidence, since 1968 until the present time. Some population based cancer registries have started but had a premature death in Latin America. Some had been too ambitious in covering too large populations or whole countries. Others have been unable to maintain its modest but much needed economic support, reflecting the shortsightedness of national and international entities that provided the initial but not the continued funding. A few had followed the Cali Registry experience and have been able to survive economic difficulties experienced recently by some Latin American countries. Hopefully their initial success can be sustained and allow them to maintain is pioneering work. Such is the case of the Cancer Registry of Quito, operating very efficiently for 25 years, and the recently created Colombian Registries in Pasto, Manizales and Bucaramanga.

The principal and most reliable sources of data are the histopathology reports. A first attempt to analyze data in a defined population was done in Medellin, when the Pathology Department of Universidad de Antioquia, founded and directed by Alfredo Correa Henao, represented the only source of histopathology diagnoses in the department of Antioquia. Such collection of data covered the years of 1944 to 1951 and was since the bases for publications in national and international journals. Later, in Cali, the histopathology diagnoses of cancer made in the pathology departments of the country were collected and presented the Third Congress of the Latin American Society of Pathology in Medellin in 1961^1^ ^,^ ^2^. Dr. Harold Stewart, head of the Department of pathology of the National Cancer Institute in Bethesda, Maryland, was a special guest of the Society at the Congress. Dr. Stewart showed special interest in the data and brought them to William Haenszel, head of the Biometry Branch of the Institute. This was the beginning of a lasting and fructiferous association of Haenszel with the Cali Registry recognized by Universidad del Valle, which gave him the title of Doctor Honoris Causa.

Dr. Haenszel, in collaboration with professors of Universidad del Valle, especially Pelayo Correa, Carlos Cuello and Guillermo Llanos, established the foundation for a Cancer Registry^3^ that produced reliable data, which have continued throughout the years by the successive Directors of the Registry: Edwin Carrascal and Luis Eduardo Bravo. Some of the bases for the operation are: to register only new cases (incidence) and include only cases of residents of the city according to the limits set by the municipality in 1962.

The Registry started at the same time that the Pan-American health Organization conducted the Study of Urban Mortality, which examined in detail all the death certificates of the city^4^. The systematic study of such certificates was part of the data collection for the Registry. All the departments of pathology of the city have generously provided the cancer diagnoses to the Registry. A system of data collection was put in place, utilizing medical students every two years, previously receiving special training. The collect data from several data sources, especially private practitioners who have collaborated in an extraordinary manner.

The Registry has been recognized by the International Association of Cancer Registries and the International Agency for Research on Cancer ( IARC) , which also provides technical support in data handling.

## Impact of the registry

Many benefits have resulted from the Registry throughout the years. It is one of the few research programs that have remained active and productive at a time when the Academic Institutions of Latin America have experienced social and economic setbacks. It has had a definite but hard to measure impact on the educational programs of the University. The students have been exposed to the importance of epidemiology on the health of the community to the point that several courses have contributed to the understanding and appreciation of its value by the teaching staff.

The Registry has called attention to the tumors that represent the main social burden, especially cancer of the cervix and the stomach. A study based on Registry data showed that the lack of vaginal cytology tests increased cervical cancer rate by a factor of 10. This led health authorities and Universidad del Valle to set-up a central cytology laboratory, under the expert direction of Dr. Nubia Aristizabal^5^to examine and report on cytology specimens collected in the health centers. Following this set-up, a marked decrease in the incidence of invasive carcinoma was reported in the following years.

The gastric cancer epidemic was recognized and was followed by a series of epidemiologic studies, especially in the rural population of Nariño, whose immigrants to Cali had the highest gastric cancer incidence rate of the population. The Nariño studies have contributed to clarify the etiology of the disease, especially the role played by the Helicobacter pylori infection and the role of dietary antioxidants. An intervention study demonstrated the benefit of controlling such etiologic factors^6^ ^,^ ^7^. 

This issue of Colombia Medica documents important advances in the generation of knowledge on the epidemiology of cancer in Colombia, a consequence of the establishment and continued functioning of several population-based Cancer Registries. Additionally, it has permitted us to analyze the time trends of tumors associated to infections: gastric and cervical cancers, documenting the end of such epidemics of neoplastic diseases. A steady increase in the frequency of cancers of the colo-rectum and female breast possibly associated with a gradual improvement of the socioeconomic conditions of the population. An unexplained increase of the incidence of thyroid carcinoma is also documented. The increase in the incidence rates of prostatic cancer possibly reflects the increased use of the prostatic specific antigen (PSA) tests that may result in false positives, tumors that lack invasive capacity. The new information should serve as a basis to continue research on this phenomenon and to design strategies to control these diseases by public health authorities.
